# Psychopathological Correlates and Emotion Regulation as Mediators of Approach and Avoidance Motivation in a Chinese Military Sample

**DOI:** 10.3389/fpsyt.2019.00149

**Published:** 2019-03-22

**Authors:** Xiaoxia Wang, Rui Zhang, Xiao Chen, Keyu Liu, Lifei Wang, Jin Zhang, Xiao Liu, Zhengzhi Feng

**Affiliations:** ^1^Department of Basic Psychology, School of Psychology, Army Medical University, Chongqing, China; ^2^Department of Behavioral Medicine, School of Psychology, Army Medical University, Chongqing, China

**Keywords:** behavioral activation/inhibition scales, depression, reinforcement sensitivity theory, factor analysis, personality psychometrics

## Abstract

**Background:** Approach and avoidance motivation have been thoroughly studied in common mental disorders, which are prevalent in the military context. Approach/avoidance motivational dispositions underlie emotion responses and are thought to influence emotion dysregulation. However, studies on the mediating role of emotion regulation (ER) between motivational dispositions and mental disorders have been insufficient. We examined the psychopathological correlates of motivational dispositions and explored the mediating role of ER.

**Methods:** The Behavioral Inhibition System and Behavioral Activation System (BIS/BAS) scales and measures of mood disorders (depression, anxiety, OCD, and PTSD) were administered to a nonclinical sample of 3,146 Chinese military service members. The Emotion Regulation Questionnaire for Armymen (ERQ-A) (Chinese version) was used to measure ER styles. We examined the reliability and construct validity of the BIS/BAS scales. Approach/avoidance motivations were correlated with symptoms of mood disorders. Mediation analysis was conducted to confirm the mediating role of ER between motivation and mood disorders.

**Results:** The results showed acceptable internal reliability and construct validity of the BIS/BAS scales. Gender (female), family status (single-parent family), and social relationships (having fewer good friends) were significant predictors of high BIS sensitivity. More years of education, an older age, being an only child and being in a single-parent family all significantly predicted high BAS sensitivity. The BIS/BAS scales were predictive of various DSM-V-based mental disorders (depression, anxiety, OCD, and PTSD). Immersion exacerbated the impact of BAS/BIS sensitivities on depressive/PTSD symptoms, while reinterpretation and talking out alleviated the impact of BAS/BIS sensitivities on these symptoms.

**Conclusions:** Motivational dispositions have an impact on mood symptoms under specific conditions. ER strategies (immersion, reinterpretation, and talking out) were shown to be partial mediators between approach/avoidance motivation and mood disorders. These findings highlight the importance of ER in altering the impact of motivational dispositions on mood disorders and as a promising target of psychotherapies.

Depression and anxiety are prevalent non-psychotic mental disorders among both American (1) and Chinese (2) military servicemen. These mental disorders are debilitating and associated with a wide range of functional impairments (e.g., decreased well-being, poorer physical health and decreased capacity for military service) (3). Substantial evidence has proposed individual differences in vulnerability to these disorders, linking motivational dispositions with specific psychopathologies, such as depression and anxiety (4–7). The approach and avoidance framework for motivational dispositions provides a parsimonious unifying framework that fits a broad variety of psychopathological disorders. Under this framework, Gray's reinforcement sensitivity theory (RST) focused on neuroanatomical models and described three major motivational systems: the Behavioral Activation System (BAS), the Behavioral Inhibition System (BIS), and the Fight-Flight System (FFS) (8), which was later revised to the FFFS (9). In the short term, these systems describe how individuals respond to motivationally significant (i.e., “reinforcing”) stimuli and which neuropsychological systems mediate these responses. For example, BAS is supposed to mediate reactions to appetitive stimuli; FFFS is supposed to mediate reactions to all (un)conditioned aversive stimuli; and BIS is responsible for the resolution of goal conflict in general. Additionally, emotion accompanies the activation of both systems and prompts pleasant or aversive affects, respectively (10). Furthermore, approach-avoidance motivation is seen as a critical and defining feature of discrete emotions (11). In the long term, individual differences in BIS and BAS sensitivity underlie the personality dimensions of anxiety and impulsivity, respectively (12). BAS may be reflective of an approach motivational disposition, while BIS/FFFS may be reflective of an avoidance motivational disposition (13). Moreover, individual differences in BIS and BAS reactivity are considered to represent stable individual differences in positive and negative affectivity.

In terms of trait manifestations of the BIS/BAS, the RST cognitive model of psychopathology assumes that individuals with extreme (either high or low) levels of BIS and BAS sensitivity are at increased risk for developing psychopathology (12), and considerable empirical evidence supports these associations. On the one hand, BAS was positively correlated with externalizing disorders (e.g., alcohol abuse, aggression) and negatively correlated with internalizing disorders (depression, anxiety) (14–16). Under-reactivity of approach motivation may underlie depression (17) and is a trait-like vulnerability marker for major depression even after recovery (18). An inverse relationship between approach motivation level and the severity of depressive symptoms was found (19). On the other hand, BIS was positively related to both depressive and anxiety disorders (4, 17, 20, 21). High BIS sensitivity has been linked to obsessive–compulsive and schizotypal personality features, while low BIS sensitivity has been linked to antisocial personality features (4, 22).

Despite the above findings, other findings suggest that the magnitude of the associations between BIS/BAS and psychopathology is at best moderate (17). Accordingly, one could plausibly infer that people with similar dispositional vulnerabilities can follow different development paths due to a variety of moderating factors. The perspective of developmental psychopathology emphasizes the influence of self-regulatory processes that enable persons to modulate emotional reactions and, as such, decrease the risk associated with this reactivity (23). Empirically, emotion (dys)regulation was proposed as a partial mediator between reinforcement sensitivity and mood symptoms such as PTSD (24) and depression (25). Specifically, individuals with high FFFS sensitivity reported higher levels of emotion dysregulation, which in turn was associated with higher PTSD scores (24). Similarly, BAS sensitivity may affect depression only under specific circumstances (e.g., in individuals low in cognitive reappraisal) (25). The specific dimensions of BAS (e.g., Drive, Fun-Seeking) demonstrated unique positive associations with adaptive ER (26). Weaker BAS-Reward Responsiveness (BASR) and stronger BIS sensitivity were related to emotion regulation difficulties (ERD) (27).

In addition, ample evidence has demonstrated a relationship between emotion regulation (ER) and mood symptoms. Meta-analyses revealed that alterations in ER capacity are common in clinical populations (e.g., patients with mood and anxiety disorders) (28). Notably, a majority of military servicemen lie in the age range of early adulthood. The ability to regulate approach/avoidance motivation depends on the maturation of regulatory networks, which persist throughout adolescence and into adulthood (29, 30). Thus, it is plausible to assume that for individuals in this age range, prefrontal development may be sensitive to imbalanced motivation systems and as such confer vulnerability to externalizing or internalizing problems (31). Therefore, additional research is necessary aiming at delineating the exact nature of the associations among BIS/BAS, ER and mental disorders.

Given these findings and the recent emphasis on the dimensional components of psychopathology (32) that might be better predictors of pathophysiology or prognosis, we need reliable and effective tools to assess individual differences in motivation components. To date, the most popular questionnaire measuring BIS and BAS sensitivity is the Behavioral Inhibition System and Behavioral Activation System scale (BIS/BAS scale, BBS) (33). Compared with other questionnaires of RST, BBS subscales (BAS-Drive [BASD], BASR, BIS) showed acceptable internal and convergent validity, except for the lack of convergent validity of the BAS-Fun Seeking (BASF) subscale (34). Although the BIS subscale conflates FFFS and BIS factors, it is not advisable to extract these two factors due to an insufficient number of items for the FFFS factor (35). Our first aim was to test the validity and reliability of the four-factor model of BBS in Chinese military men; this aim will also allow an evaluation of the BBS's cross-language validity.

The second aim was to test the hypothesis that subscales of BBS could predict mood symptoms (depression and anxiety) in Chinese servicemen. Previous evidence demonstrated that depressed deployed veterans have higher rates of comorbid anxiety and PTSD disorders than depressed non-deployed veterans (36). Soldiers with PTSD and an internalizing personality profile (low extraversion, which was closely related to low BAS) are more likely to experience internalizing comorbidity (e.g., anxiety, depression) (37). In consideration of the high comorbidity of PTSD and other disorders (anxiety and depression), we also focused on PTSD symptoms.

Due to the evidence that ER may either alleviate depressive symptoms (38), posttraumatic symptoms (39), or anxiety symptoms (40), our third aim was to test the mediating effect of ER in predicting mood symptoms (depression, anxiety, PTSD) from approach/avoidance motivation. The Emotion Regulation Questionnaire for Armymen (ERQ-A) was used because it was specifically developed for Chinese military men and measures the cognitive ER strategies they habitually use.

## Method

### Participants

This research was reviewed and approved by the Human Research Ethics Committee of Army Medical University before the study began. The participants were 3,146 military men (age: 21.55 ± 3.05; service years: 3.24 ± 3.45) based on cluster sampling from June through December 2014. In view of the large sample size and the time limitation of the population under survey, Verbal informed consent was obtained before the participants completed the survey. However, they were instructed to be free to withdraw from the study at any time and their private information provided would be kept confidential. The verbal informed consent process was approved by the ethics committee of the university. The documentation of the consent process including the names of all participants, information provided and date consent obtained was kept in the study record. To enhance their understanding and recall of the information given by the investigators, all participants were also informed of the aim of the study. The demographic characteristics of the participants are listed below (Table 1). Participants were randomly separated into two groups of equal numbers for exploratory factorial analysis (EFA) and confirmatory factorial analysis (CFA). The proportions of gender, family status, only child status, and education years were homogeneous between these two groups (*P* > 0.05).

**Table 1:** Demographic characteristics of Chinese military men sample.

**Name**	**Frequency, *N***	**Percentage (%)**
**GENDER**		
Male	2,949	93.74
Female	160	5.09
Missing value	32	1.17
**ONLY CHILD**		
Yes	1,109	35.25
No	1,971	62.65
Missing value	65	2.10
**COMMISSIONED RANK**		
Soldier	2,111	67.10
Sergeant	884	28.10
Officer	99	3.15
Missing value	52	1.65
**EDUCATION**		
Middle school or below	552	17.55
High school	1,742	55.37
College degree	562	17.86
Undergraduate	238	7.57
Postgraduate	5	0.16
Missing value	47	1.49
**FAMILY STRUCTURE**		
Two-parent	2,717	86.36
Single-parent (with mother)	144	4.58
Single-parent (with father)	145	4.61
Other type	93	2.96
Missing value	43	1.49

### Measurements

#### Behavioral Activation/Inhibition Scales

The BBS (33) was translated into Chinese and revised in a sample of Chinese medical college undergraduate students (42). The Chinese undergraduate BBS includes 18 items (item 1/18 deleted), and four filler questions were not used in calculating subscale scores. A previous study of Chinese undergraduate students found that their data adequately fit the four-factor model (42). The original BBS Cronbach's α was 0.66~0.76, and the revised Chinese undergraduate BBS Cronbach's α was 0.55~0.72 (Table 2). The original BBS test-retest reliability (8 weeks apart) was 0.59~0.69 (33). The four subscales of BBS were BIS, BASR, BASD, and BASF. The subscales used a 4-point Likert scale for response options (e.g., 1 = Strongly Agree; 2 = Somewhat Agree; 3 = Somewhat Disagree; 4 = Strongly Disagree). Reward Responsiveness, Drive and Fun Seeking were used to measure BAS dispositions. The BASR subscale measures the tendency to respond with positive affect to desired events or cues of potential future reward. The BASD subscale emphasizes motivation to pursue goals, regardless of whether these goals are inherently pleasurable. The BASF subscale emphasizes the impulsive pursuit of pleasure.

**Table 2:** Internal consistency and item-total score Pearson correlations for different versions of Behavioral Activation/Inhibition scales (BBS).

**Subscale**	**Cronbach's** **α**				**Item-total Pearson correlations**		
	**American adults (41)**	**Chinese undergraduates (42)**	**Chinese military men**		**Chinese undergraduates (42)**	**Chinese military men**	
			**Original^b^**	**4-factor^c^**		**Original**	**4-factor**
BIS	0.77	0.59	0.76	0.65	–	0.30~0.74^**^	0.75~0.78^**^
Drive	0.85	0.66	0.73	0.71	–	0.36~0.79^**^	0.76~0.83^**^
RR	0.89	0.72	0.60	0.73	–	0.24~0.73^**^	0.67~0.78^**^
FS^a^	0.80	0.55	0.72	0.75	–	0.23~0.74^**^	0.73~0.79^**^
Full scale	–	0.70	0.81	0.85	0.13~0.54^**^	0.11~0.78^**^	0.44~0.67^**^

*BIS, Behavioral Inhibition Scale; Drive, BAS-Drive scale; RR, BAS-Reward Responsiveness scale; FS, BAS-Fun Seeking scale*.

** *P < 0.01*.

a *The original examination of BBS was conducted on the full sample and was based on the four-factor model of Chinese undergraduate version of BBS. The revision was conducted on the random half-sample, which yielded the four-factor model of Chinese military men*.

b *The Chinese undergraduate 4-factor model was fitted to the data set of Chinese military men*.

c *The Chinese military men 4-factor model was revised based on the Chinese undergraduate 4-factor model*.

#### Mood Symptoms

The Symptom Checklist-90-Revised (SCL-90-R) Depression Scale is a widely applied self-assessment instrument for a broad range of subjective symptoms resulting from mental disorders. The SCL-90 contains 9 subscales: Somatization, Obsessive-Compulsive, Depression, Anxiety, Hostility, Phobic Anxiety, Paranoid Ideation, and Psychoticism. The depression dimension includes 13 items (5, 14, 15, 20, 22, 26, 29, 30, 31, 32, 54, 71, and 79). The anxiety dimension includes 10 items (2, 17, 23, 33, 39, 57, 72, 78, 80, and 86). The obsessive-compulsive dimension includes 10 items (3, 9, 10, 28, 38, 45, 46, 51, 55, and 65). The Chinese version of SCL-90-R showed good validity (43). Based on a larger sample that was utilized to establish the norm (2016 Edition) of SCL-90 for Chinese military personnel (2), the current study mainly focused on the depression and anxiety dimensions.

The Posttraumatic Stress Disorder (PTSD) Checklist (PCL-M) (military version) is a 17-item self-report measure of the DSM-IV symptoms of PTSD and can be used for screening and diagnosis of PTSD. The PCL-M measures symptoms in response to “stressful military experiences” and is used for active service members and veterans. All of the rating scale descriptors are the same: “Not at all,” “A little bit,” “Moderately,” “Quite a bit,” and “Extremely.” A total score of 50 is considered PTSD positive in military populations. Internal consistency coefficients were very high for the total scale 0.97) and for each subscale (0.92–0.93). The test-retest reliability over 2~3 days was 0.96 (44).

#### Emotion Regulation Questionnaire for Armymen

The Emotion Regulation Questionnaire for Armymen (ERQ-A) was developed based on a cluster sample of 1,738 Chinese armymen and measures the cognitive ER strategies they habitually use. This questionnaire is available only in a Chinese language version and includes four subscales: immersion, reinterpretation, talking out and expression suppression. Immersion is the process of being immersed in the experienced emotion through emphasizing the emotional salience and self-relevance of the emotion-eliciting events. This strategy may enhance the person's emotions because imagining increased self-involvement with emotion-eliciting events requires close attention to and elaboration of the linguistic content of those events (45). Reinterpretation requires the individual to distance himself from the experienced emotion through reappraising the emotional salience and self-relevance of the emotion-eliciting events. Talking out enables the individual to seek help from others and confide their concealed emotions to alleviate the experienced emotion. Expression suppression is the process of inhibiting already ongoing emotion-expressive behavior, similar to the expression suppression proposed by Gross (46).

The Cronbach's α coefficients of the total scale and subscales were 0.875 and 0.680~0.769, respectively. The split-half reliability values of the total scale and subscales were 0.854 and 0.110~0.791, respectively. The correlation coefficients between total score and factors were 0.656~0.791, and the correlation coefficients among factors were 0.110 0.791. Additionally, 42.86% of the items in the questionnaire had a high discrimination index (D ≥ 0.4) (47). All four types of ER habitual use were predictors of depression, which could explain 35.5% of the total variance of depressive symptoms. The risk factor was immersion, and the protective factors were reinterpretation, expression suppression and talking out. ER tendencies are important predictive factors of depression in Chinese armymen (48).

### Statistical Analysis

#### Reliability and Validity Analysis of the BIS/BAS Scales

Data analysis was conducted using SPSS software version 22.0 for Windows (SPSS Inc., Chicago, IL, USA). The content validity was assessed using item-total correlation and internal consistency (Cronbach's alpha). To determine the number of factors to extract for exploratory factor analysis (EFA), we first fixed the number of factors according to the four-factor model and then used a rule of thumb that retains all factors with an eigenvalue >1. Specifically, items that showed strong loadings exclusively on a single factor (≥0.45) were retained in the model. Items that showed weak loadings (<0.4) on all factors or cross-loadings (≥0.45) onto multiple factors were deemed problematic and were deleted. Oblique rotations (the promax rotation) were used because there was prior evidence that factor inter-correlations of BBS exceeded 0.3 (49). All the resulting models were later tested using confirmatory factor analysis (CFA), which was conducted with Amos 17.0 software. The χ^2^, comparative fit index (CFI), and root mean squared error of approximation (RMSEA) with the associated 90% confidence interval (CI) and *P*-values were utilized to assess the goodness of model fit.

#### Relationships Between BIS/BAS Sensitivities and Mood Symptoms

We fist evaluated the significance of BIS and BAS levels as predictors of the occurrence of one or two internalizing symptoms (depression, anxiety) via a multivariate analysis of variance (MANOVA) (see section Results in Approach/Avoidance Motivation as Risk Factors for the Occurrence of One or Moremood Symptoms). The dependent variables were BIS, BASR, BASD, and BASF. To estimate the significance of BIS and BAS for specific forms of comorbidity, the independent variables captured the presence or absence of the mood symptoms of (a) depression, (b) anxiety, and (c) their interaction terms. For example, an interaction between depression and anxiety would suggest that the subscales of BBS have unique implications for understanding the comorbidity of depression and anxiety symptoms compared to depression or anxiety symptoms alone. Wilk's lambda was used to estimate the multivariate *F* [if assumed variance homogeneity was satisfied (Levene's test: *P* < 0.001); otherwise, Pillai's trace was used]. To control for the confounding effects of participants' demographic variables (e.g., age, gender, length of service, family status, and social relationships), we included these variables as covariates in the MANOVA analysis.

To examine significant multivariate effects, univariate analysis of variance (ANOVA) was then computed for each dependent variable: BIS, BASR, BASD, and BASF (see Section Results in Approach/Avoidance Motivation as Risk Factors for the Occurrence of One or Moremood Symptoms). To provide another index of the magnitude of significant effects, a logistic regression analysis was performed, and odds ratios were calculated for each significant effect (see section results in Approach/Avoidance Motivation as Risk Factors for the Occurrence of One or Moremood Symptoms). Participants scoring within the upper 25% or lower 25% of the BIS or BAS subscale scores were categorized into high/low BIS (or BAS) groups. To avoid inflating type II error, these analyses are included only for the main effects that were significant within MANOVAs. The odds ratios of depression and anxiety were calculated taking comorbidity into account.

We also compared participants with higher vs. lower levels of internalizing symptoms via *t*-tests and estimated correlations of BIS/BAS scale scores with depressive and anxiety symptoms (see section Results in Relationships Between BIS/BAS Scores and Severity of Mood Symptoms). Significant correlations were considered support for predictive validity, as these correlations would be hypothesized.

#### Mediating Effects of ER Between BIS/BAS Sensitivity and Mood Symptoms

We conducted mediation analysis using the bootstrapping method with depressive or PTSD symptoms as the dependent variable, BAS/BIS as the independent variable, and frequent use of ER as each mediating variable. The bootstrapping test for mediation analysis was conducted using the PROCESS mediation macro in SPSS (model 4) (50), and 95% CIs of indirect effects were reported while making no assumptions about the distribution of indirect effects.

## Results

### Internal Consistency Reliability and Construct Validity of BIS/BAS Scales

The average scores on 18 items were 2.33~3.38 (standard deviation 0.69~0.90). We analyzed the internal consistencies and item-total Pearson correlations of BBS based on the Chinese military sample. The internal consistencies of BIS, BASD, and the full scale were greater in the Chinese military men sample compared with Chinese undergraduates. Additionally, the internal consistency of the total scale and the item-total Pearson correlations was greater than that of the original scale (Table 2).

The results showed perfect adequacy of the data for factorial analyses (KMO = 0.91; Bartlett's sphericity test, *df* = 153, *P* < 0.001). Because the Pearson correlations between each two factors exceeded 0.3, promax rotation was used. According to the criterion that communities were >0.2 for items and factor loadings were >0.45 after rotations, four factors were extracted, which explained 54.784% (cumulative%) of the total variance. Several items (13/16/8/7/11/10/3) were reassigned to another subscale. The factor loadings for items 4/6/8/15/10/3/9/18/12/14 were equal to or greater than those of the original model in the Chinese undergraduate sample (Table 3).

**Table 3:** Exploratory factorial analysis of Behavioral Activation/Inhibition scales of Chinese military men.

**Item**	**Factor (Chinese undergraduate sample)**	**Factor loadings**	**Factor (Chinese military sample)**	**Factor loadings**
2. When I'm doing well at something I love to keep at it.	BASR	0.75	BASR	0.72
4. When I get something I want, I feel excited and energized.	BASR	0.73	BASR	0.74
17. It would excite me to win a contest.	BASR	0.72	BASR	0.58
13. When good things happen to me, it affects me strongly.	BASR	0.72	BIS	0.67
1. I go out of my way to get things I want.	BASD	0.76	BASD	0.71
16. When I go after something I use a “no holds barred” approach.	BASD	0.76	BASF	0.67
6. When I want something I usually go all-out to get it.	BASD	0.62	BASD	0.82
8. If I see a chance to get something I want I move on it right away.	BASD	0.61	BASR	0.61
7. I'm always willing to try something new if I think it will be fun.	BASF	0.67	BASD	0.66
11. I will often do things for no other reason than that they might be fun.	BASF	0.64	BIS	0.59
15. I often act on the spur of the moment.	BASF	0.63	BASF	0.68
10. I crave excitement and new sensations.	BASF	0.50	BASR	0.68
3. When I see an opportunity for something I like I get excited right away.	BASF	0.42	BASR	0.67
5. Criticism or scolding hurts me quite a bit.	BIS	0.67	BIS	0.57
9. I feel pretty worried or upset when I think or know somebody is angry at me.	BIS	0.65	BIS	0.67
18. I worry about making mistakes.	BIS	0.59	BIS	0.69
12. I feel worried when I think I have done poorly at something important.	BIS	0.46	BIS	0.77
14. If I think something unpleasant is going to happen I usually get pretty “worked up.”	BIS	0.43	BIS	0.74

*BASD, BAS-Drive scale; BASF, BAS-Fun seeking scale. BASR, BAS-Reward Responsiveness scale. BIS, Behavioral Inhibition Scale. The Chinese undergraduate 4-factor model was applied to the data set*.

In consideration of the cutoff criteria (CFI ≥ 0.90, RMSEA ≤ 0.08), the Chinese undergraduate four-factor model conducted on Chinese military men showed a relatively poor fit (CFI = 0.56, RMSEA = 0.13). In contrast, the Chinese military four-factor model achieved acceptable model fit (CFI = 0.94, between 0.90 and 0.95) and moderate model fit (RMSEA = 0.06, between 0.05 and 0.08). Moreover, the improved model fit was similar to that of a measurement of American adults (CFI = 0.96, RMSEA = 0.07) (41) (Table 4). Due to sensitivity to sample size and violations of normality assumptions, the chi-squared test was not considered for measuring model fit (49).

**Table 4:** Summary of factor analyses for versions of revised Behavioral Activation/Inhibition scales.

**Sample**	**Age**	**Sample**	**EFA**			**CFA**				
	**($\bar{\text{x}}\;\pm S\text{D}$)**	**size**	**KMO**	**Explained variance%**	**Deleted items**	**Factor**	**χ^2^**	***df***	**CFI**	**RMSEA**
American young adults (49)	19.30 ± 1.01	844	–	–	3/7/10/11/ 14/15/18	3	69.96^*^	51	0.99	0.03
American adults (41)	21~40	631	–	–	–	4	610^*^	–	0.96	0.07
Chinese undergraduates (42)	20 ± 2	262	0.72	45.16	1/18	4	196.35^**^	129	0.89	0.04
Chinese military men (Chinese undergraduate model ^a^)	21.42 ± 2.71	1,573	0.91	54.49	1/18	4	3,840.70^**^	135	0.56	0.13
Chinese military men (4-factor)	21.42 ± 2.71	1,573	0.90	57.43	7/17/15/8	4	429.97^**^	71	0.94	0.06

*The means and standard deviation of the sample were not reported. The American young adult version of BBS contained 24 items, and the Chinese undergraduate version comprised 18 items*.

a *We tested the Chinese undergraduate four-factor model in military personnel and then revised it to the four-factor model of Chinese military men according to the results of EFA and CFA*.

* *P < 0.05*;

** *P < 0.01*.

### Influence of Demographic Characteristics on Approach/Avoidance Motivation

#### BIS

Step-wise multiple regression analyses revealed that gender (beta = 0.102, *P* < 0.001), family status (beta = 0.056, *P* = 0.003) and social relationships (beta = 0.044, *P* = 0.019) significantly predicted BIS levels. Specifically, (1) female military members showed greater levels of BIS (female: 14.513 ± 2.853; male: 13.216 ± 2.944) (*t* = −5.400, *P* < 0.001). (2) Those participants with a single parent (living with father) showed the greatest levels of BIS among the three types of family status [both parents: 13.239 ± 2.933; single parent (with mother): 13.333 ± 2.833; single parent (with father): 13.951 ± 3.251] (*F* = 2.436, *df* = 4, *P* = 0.045), significantly greater than those participants living with a single parent (mother) (*post-hoc* comparison and LSD correction: *t* = 0.712, *P* = 0.005). (3) Those participants having no good friends showed the greatest levels of BIS (having no good friends: 13.236 ± 2.948; having 1~2 good friends: 13.775 ± 2.812; having 3~5 good friends: 14.180 ± 3.852) (*F* = 4.068, *df* = 3, *P* = 0.007), significantly greater than those having 3~5 good friends (*post-hoc* comparison and LSD correction: *t* = 0.943, *P* = 0.047) (Table 5).

**Table 5:** Effects of demographic characteristics on approach/avoidance motivation.

**BBS factors**	**Demographic variables**	***Post-hoc* comparison**	**Beta (Pearson r), Sig**.
BIS	Gender	Female > male	Beta = 0.102, *P* < 0.001
	Family status	Single parent (with father) > single parent (with mother) > both parents	Beta = 0.056, *P* = 0.003
	Social relationships	Having no good friends > having 1~2 friends > having 3~5 friends	Beta = 0.044, *P* = 0.019
BASD	Education years	Middle school = high school < junior college = bachelor's degree	Beta = 0.077, *P* < 0.001
	Only child status	Non-only child < only child	Beta = −0.050, *P* = 0.008
	Gender	Female > male	Beta = −0.046, *P* = 0.017
	Age		Beta = −0.042, *P* = 0.031
			*r* = −0.039, *P* = 0.037
BASR	Education years	Middle school < high school < junior college = bachelor's degree	Beta = 0.101, *P* < 0.001
	Commissioned/ non-commissioned rank	Officers = soldiers > sergeants	Beta = −0.050, *P* = 0.010
BASF	Age		Beta = −0.098, *P* < 0.001
			*r* = −0.098, *P* < 0.001
	Only child status	Non-only child < only child	Beta = −0.064, *P* = 0.001
	Education years	Middle school < high school = bachelor's degree = junior college	Beta = 0.068, *P* < 0.001
	Family status	Single parent (with father) = single parent (with mother) > both parents	Beta = 0.043, *P* = 0.021

#### BASD

Step-wise multiple regression analyses revealed that education years (beta = 0.077, *P* < 0.001), only child status (beta = −0.050, *P* = 0.008), gender (beta = −0.046, *P* = 0.017) and age (beta = −0.042, *P* = 0.031) significantly predicted BASD levels. Specifically, (1) participants who had a middle school education showed the lowest BASD levels (*F* = 2.735, *df* = 4, *P* = 0.027), significantly different from participants with other education status. (2) The participants who were only children showed greater BASD levels than non-only children (*t* = 2.778, *P* = 0.005). (3) Partial correlation of BASD and age (controlling for the effects of other demographic variables) yielded non-significant negative associations (*r* = −0.032, *P* = 0.076). (4) BASD levels yielded significant differences between male and female military members (*t* = 1.763, *P* = 0.038), with greater BASD for female members (Table 5).

#### BASR

Step-wise multiple regression analyses revealed that education years (beta = 0.101, *P* < 0.001) and commissioned/non-commissioned rank (beta = −0.050, *P* = 0.010) significantly predicted BASR levels. Specifically, (1) participants who had a middle school education showed the lowest BASR levels (*F* = 5.923, *df* = 4, *P* < 0.001), significantly different from participants with other education status (middle school education < high school education < junior college education = bachelor's degree education). (2) Officers had the greatest levels of BASR (*F* = 4.833, *df* = 2, *P* = 0.008), similar to soldiers (*post-hoc* comparison and LSD correction: *t* = 0.250, *P* = 0.214) and higher than sergeants (*post-hoc* comparison and LSD correction: *t* = 0.461, *P* = 0.026) (Table 5).

#### BASF

Step-wise multiple regression analyses revealed that age (beta = −0.098, *P* < 0.001), only child status (beta = −0.064, *P* = 0.001), education years (beta = 0.068, *P* < 0.001) and family status (beta = 0.043, *P* = 0.021) significantly predicted BASF levels. Specifically, (1) partial correlation of BASF and age (controlling for the effects of other demographic variables) yielded significant negative associations (*r* = −0.071, *P* < 0.001). (2) Participants who were only children showed greater BASF levels than non-only children (*t* = 4.083, *P* < 0.001). (3) Participants who had a middle school education showed the lowest BASF levels (*F* = 4.396, *df* = 4, *P* = 0.002), significantly different from participants with other education status. (4) Participants who lived with a single parent (father) showed the greatest BASF levels (*F* = 4.429, *df* = 4, *P* = 0.046), similar to participants living with a single parent (mother) (*post-hoc* comparison and LSD correction: *t* = 0.493, *P* = 0.133) and higher than participants living with both parents (*post-hoc* comparison and LSD correction: *t* = 0.587, *P* = 0.014) (Table 5).

### Approach and Avoidance Motivation as Predictors of Mood Symptoms

The results of MANOVAs, ANOVAs and the logistic regression model confirmed our hypotheses that BIS and BAS are associated with a broad range of psychopathologies.

#### Approach/Avoidance Motivation as Risk Factors for the Occurrence of One or More Mood Symptoms

For the constellations of BBS subscales as the dependent variables of MANOVA, main effects of depression, anxiety, and PTSD were significant when taking depression, anxiety, obsessive-compulsion, and PTSD as independent variables (Table 6). Similar results were yielded when controlling for the confounding effects of demographic variables. Furthermore, to examine the main and interaction effects of depression and anxiety with other comorbidities, we conducted the following analyses: (1) When taking depression and anxiety as independent variables, the main effect of depression was significant (*P* = 0.005, η^2^ = 0.029); (2) when taking depression, anxiety and obsessive-compulsion as independent variables, the main effects of depression (*P* = 0.029, η^2^ = 0.022) and obsessive-compulsion (*P* = 0.028, η^2^ = 0.039) were significant. Interaction between depression and anxiety (*P* = 0.011, η^2^ = 0.073) significantly predicted BIS and BAS levels. Interaction between depression and obsessive-compulsion (*P* = 0.010, η^2^ = 0.105) significantly predicted BIS and BAS levels.

**Table 6:** Multivariate and univariate analyses of variance for BIS and BAS subscales by the symptoms of depression, anxiety, OCD, and PTSD.

**Effect**	**Multivariate**		**Univariate effects (*****F*****)**			
	***F***	*****η^2^*****	**BIS**	**Drive**	**Reward**	**Fun seeking**
Depression	3.785^**^	0.005	11.910^**^	1.766	0.023	5.541^*^
Anxiety	4.004^**^	0.005	0.530	0.041	0.045	0.090
OCD	0.361	0.000	1.272	0.689	2.937	1.308
PTSD	3.582^**^	0.005	14.289^***^	6.397^*^	1.676	12.928^***^
Depression × Anxiety	–	–	–	–	–	–
Depression × OCD	1.145	0.002	0.029	0.516	1.717	0.501
Depression × PTSD	2.360	0.003	0.090	0.281	4.283^*^	1.719
Anxiety × OCD	1.095	0.001	1.185	0.020	0.078	0.036
Anxiety × PTSD	1.555	0.002	4.281^*^	2.009	0.027	1.231
OCD × PTSD	0.717	0.001	0.169	0.299	0.865	0.716
Depression × Anxiety × OCD	–	–	–	–	–	–
Depression × Anxiety × PTSD	–	–	–	–	–	–
Depression × Anxiety × OCD × PTSD	–	–	–	–	–	–
Depression × OCD × PTSD	–	–	0.025	2.344	2.075	1.072
Anxiety × OCD × PTSD	0.464	0.001	–	–	–	–

*Depression, anxiety, obsessive-compulsion and PTSD were entered as independent variables. Depression, displaying depressive symptoms; Anxiety, displaying anxiety symptoms; OCD, displaying obsessive-compulsive symptoms; PTSD, displaying PTSD symptoms*.

* *P < 0.05*;

** *P < 0.01*;

*** *P < 0.001. – Inapplicable due to insufficient cases*.

For either BIS or BAS sensitivity as the dependent variable of univariate ANOVA, depression (*F* = 11.910, *df* = 1, *P* = 0.001) and PTSD (*F* = 14.289, *df* = 1, *P* < 0.001) showed significant main effects on BIS levels, and PTSD (*F* = 6.397, *df* = 1, *P* = 0.011) showed a significant main effect on BASD levels. Depression (*F* = 5.541, *df* = 1, *P* = 0.019) and PTSD (*F* = 12.928, *df* = 1, *P* < 0.001) showed significant main effects on BASF levels (Table 6).

**Figure 1:**
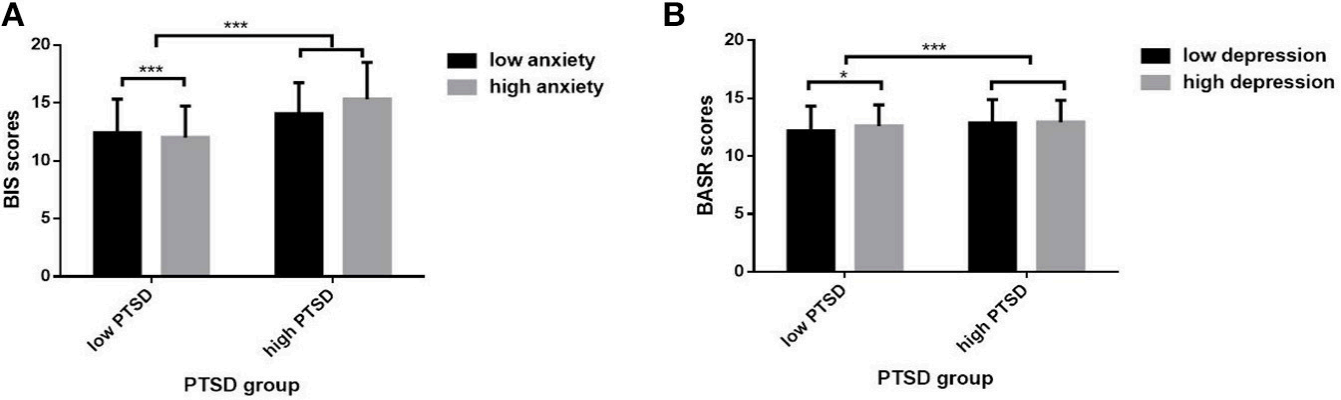
Interaction effects of mood symptoms on BIS/BAS sensitivities. **(A)** The interaction effect between PTSD and anxiety on BIS level. **(B)** The interaction effect between PTSD and depression on BASR level. *P < 0.05, ***P < 0.001.

Specifically, the interaction between PTSD and anxiety (*F* = 4.281, *df* = 1, *P* = 0.039) showed a significant effect on the BIS level. The interaction between PTSD and depression (*F* = 4.283, *df* = 1, *P* = 0.039) showed a significant effect on the BASR level. *Post-hoc* comparisons revealed that the low- vs. high-anxiety groups with fewer PTSD symptoms differed in BIS levels (*t* = 5.969, *P* < 0.001), while the low- vs. high-depression group with fewer PTSD symptoms differed in BASR levels (*t* = 2.555, *P* = 0.011; Figures 1A,B).

Logistic regression analysis of each BBS subscale (BIS/BAS) confirmed the above MANOVA and ANOVA results, with anxiety, depression and PTSD scores as independent variables. Greater depression (β = 0.090, *P* < 0.001) and PTSD (β = 0.083, *P* < 0.001) symptoms were predictive of higher BIS levels. Greater depression (β = 0.027, *P* = 0.013) and PTSD (β = 0.035, *P* < 0.001) symptoms were predictive of higher BASD levels. Greater PTSD (β = 0.052, *P* < 0.001) symptoms were predictive of higher BASR levels. Greater PTSD (β = 0.113, *P* < 0.001) symptoms were predictive of higher BASF levels.

#### Relationships Between BIS/BAS Scores and Severity of Mood Symptoms

##### Depressive symptoms

The scores on BIS (*t* = −15.006, *P* < 0.001), BASD (*t* = −6.048, *P* < 0.001), BASR (*t* = −6.686, *P*<*0.001*) and BASF (*t* = −12.136, *P* < 0.001) were significantly greater for participants with greater depressive symptoms (depression factor score ≥2). For the low-depressive group, no significant correlations between BBS subscale scores and anxiety symptoms were found. For the high-depressive group, the higher were the depressive symptoms, the higher was the BIS, BASD or BASF (Table 7).

**Table 7:** Correlations between BIS/BAS subscale scores and severity of mood symptoms.

**Motivations**	**Affective symptoms**							
	**Depression**		**Anxiety**		**OCD**		**PTSD**	
	**Low**	**High**	**Low**	**High**	**Low**	**High**	**Low**	**High**
BIS	−0.051	0.251^***^	0.162^***^	0.121	0.142^***^	0.114^***^	0.247^***^	−0.159
BASD	–	0.117^***^	0.022	0.135	0.028	0.132^***^	0.121^***^	0.117
BASR	−0.065	0.049	0.039	−0.078	0.057^*^	−0.002	0.114^***^	−0.101
BASF	−0.073	0.182^***^	0.080^***^	0.074	0.107^***^	0.171^***^	0.220^***^	0.085

*** *P < 0.001*;

* *P < 0.01 (Bonferroni corrected); – inapplicable because at least one of the variables was a constant*.

##### Anxiety symptoms

The scores on BIS (*t* = −7.662, *P* < 0.001), BASD (*t* = −5.204, *P* < *0.001*) and BASF (*t* = −5.946, *P* < *0.001*) were significantly greater for participants with anxiety symptoms (anxiety factor score ≥2). For the low-anxiety group, the higher were the anxiety symptoms, the higher was the BIS and BASF. For the high-anxiety group, no significant correlations between BBS subscale scores and anxiety symptoms were found (Table 7).

##### OCD symptoms

The scores on BIS (*t* = −18.617, *P* < 0.001), BASD (*t* = −6.566, *P* < *0.001*), BASR (*t* = −8.560, *P* < *0.001*) and BASF (*t* = −13.023, *P* < *0.001*) were significantly greater for participants with greater obsessive-compulsive symptoms (OCD factor score ≥2). For the low-OCD group, the higher were the OCD symptoms, the higher were the BIS, BASR and BASF. For the high-OCD group, the higher were the OCD symptoms, the higher were the BIS, BASD and BASF (Table 7).

##### PTSD symptoms

The scores on BIS (*t* = −17.017, *P* < 0.001), BASD (*t* = −7.851, *P* < *0.001*), BASR (*t* = −7.370, *P* < *0.001*) and BASF (*t* = −15.288, *P* < *0.001*) were significantly greater for participants with greater PTSD symptoms (≥the group mean scores). For the low-PTSD group (with a cutoff score of 50), significant correlations between PTSD and each BBS subscale score were found. For the high-PTSD group, no significant correlations between PTSD and each BBS subscale score were found (Table 7).

### Mediation Effect of ER Between Approach/Avoidance Motivation and Internalizing Symptoms

**Figure 2:**
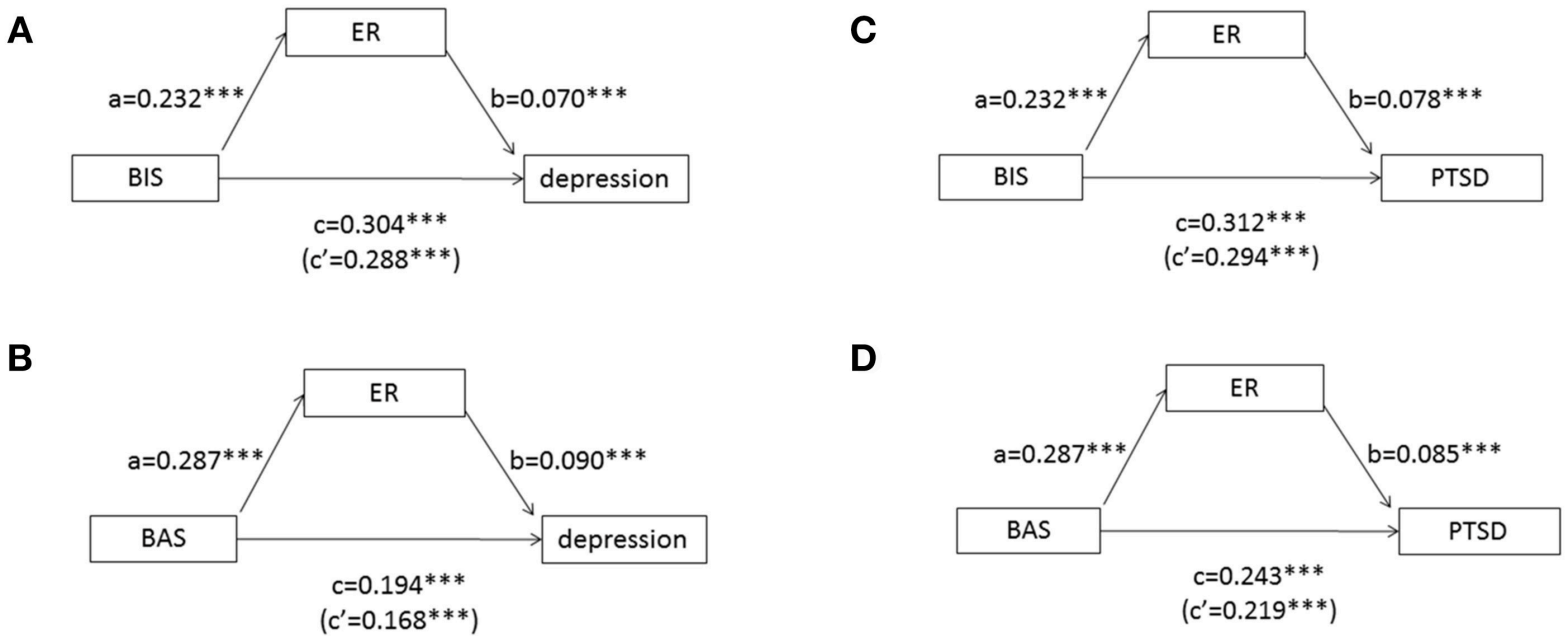
Hypothesized meditation models between motivation, emotion regulation, and mood symptoms. **(A)** Emotion regulation (ER) partially mediates the relationship between BIS sensitivity (as measured by the BBS) and depression (as measured by the SCL-90). **(B)** ER partially mediates the relationship between BAS sensitivity (as measured by the BBS) and depression. **(C)** ER partially mediates the relationship between BIS sensitivity and PTSD (as measured by the PCL-M). **(D)** ER partially mediates the relationship between BAS sensitivity and PTSD. Path values represent standardized regression coefficients (unstandardized coefficients are presented in the text). Bootstrapping and causal path analyses suggest mediation in both models. ***P < 0.001.

To test whether ER deficits mediate the relationship between motivation and mood symptoms, we used (1) the causal steps method (51) and (2) non-parametric bootstrapping using 10,000 resamples (52). For the dependent variables, we included only the mood symptoms with significant main effects for MANOVAs or ANOVAs. In the causal steps method, we used linear regressions to determine whether (1) motivation was related to mood symptoms (path c), (2) motivation was related to ER (path a), (3) ER was related to mood symptoms (path b), and (4) the standardized regression coefficients predicting mood symptoms by motivation decreased when ER was entered separately from motivation (path c'). Standardized regression coefficients for the multiple mediation models are depicted below (Figure 2). In addition, taking the ratio of BAS to BIS as the independent variable in the linear regression model also confirmed the role of ER as a partial mediator between approach/avoidance ratio and mood symptoms. In the bootstrapping estimation method, the statistical significance of each indirect pathway from ER to depressive/PTSD symptoms was examined. If the 95% CI of an estimated effect did not include zero, this effect was considered significant at the 5% level (52).

Both BAS and BIS had a direct effect on depressive symptoms (estimate of the direct effect: 0.065, 0.279) (Figure 2A). Mediation model 1 (BAS->ER->depression) explained 34.46% of the variance of depressive symptoms. Mediation model 2 (BIS->ER->depression) explained 43.19% of the variance of depressive symptoms. Specifically, immersion (*B* = 0.137; SE = 0.012; 95% CI = 0.115–0.160), reinterpretation (*B* = −0.017; SE = 0.004; 95% CI = −0.026~-0.010) and talking out (*B* = −0.014; SE = 0.004; 95% CI = −0.022~-0.008) significantly mediated the relationship between BAS and depressive symptoms. Immersion (*B* = 0.320; SE = 0.025; 95% CI = 0.274~0.371) and talking out (B = −0.023; SE = 0.007; 95% CI = −0.040~-0.012) significantly mediated the relationship between BIS and depressive symptoms. Taking ER as one mediating variable between motivation and depression confirmed its indirect effect (BAS as independent variable: *B* = 0.023; SE = 0.005; 95% CI = 0.015~-0.035; BIS as independent variable: *B* = 0.313; SE = 0.008; 95% CI = 0.016~0.048).

Both BAS and BIS had a direct effect on PTSD symptoms (estimate of the direct effect: 0.157, 0.400, *P* < 0.001) (Figure 2B). Mediation model 5 (BAS->ER->PTSD) explained 50.69% of the variance of PTSD symptoms. Mediation model 6 (BIS->ER->PTSD) explained 43.19% of the variance of PTSD symptoms. Specifically, immersion (*B* = 0.217; SE = 0.016; 95% CI = 0.186~0.249), reinterpretation (*B* = −0.019; SE = 0.005; 95% CI = −0.030~-0.009) and talking out (B = −0.029; SE = 0.006; 95% CI = −0.041~-0.018) significantly mediated the relationship between BAS and PTSD symptoms. Immersion (B = 0.531; SE = 0.037; 95% CI = 0.463~0.608) and talking out (*B* = −0.047; SE = 0.013; 95% CI = −0.076~-0.026) significantly mediated the relationship between BIS and PTSD symptoms. Taking ER as one mediating variable between motivation and PTSD confirmed its indirect effect (BAS as independent variable: *B* = 0.033; SE = 0.008; 95% CI = 0.021~-0.052; BIS as independent variable: *B* = 0.052; SE = 0.013; 95% CI = 0.029~0.078).

## Discussion

Overall, the findings support and extend previous work in this area and support the use of BBS as a powerful predictor of more global measures of internalizing symptoms. This study broadens existing knowledge by investigating the effects of BIS/BAS and ER on various mood symptoms based on DSM-IV dimensions.

### Reliability and Validity of the Chinese Military Men Four-Factor Model of Behavioral Inhibition/Activation

The internal consistencies of the subscales of the Chinese military men (revised) version of BBS were greater than those of Chinese undergraduate students but no greater than those of American adults (41). The construct validity analysis in our sample did not confirm the Chinese undergraduate four-factor model. The revised four-factor model (Fun Seeking, Reward Responsiveness, Drive, and Behavioral Inhibition) exhibited better model fit indices than the Chinese undergraduate four-factor model (42) and the American adult four-factor model (41).

#### BASR

Compared with Fun Seeking, Reward Responsiveness is more concerned with positive emotional responses to the occurrence or anticipation of reward (33). In line with this conceptualization, our revised RR subscale measured not only hedonic aspects (item 2/4/17) but also motivational aspects (item 3), corresponding to the BAS-liking and BAS-capture latent variables of BAS (34). Item 10, “When I see an opportunity for something I like I get excited right away,” belonged to the RR subscale in the original English version (33). However, this item was ascribed to the FS subscale in the Chinese undergraduate version (42). This item was reallocated to the RR subscale in our revised version of the Chinese military-men four-factor model. People high in Reward Responsiveness are motivated by rewards, and people high in fun seeking are motivated to seek out new experiences, which resembles sensation/novelty seeking (53). Although “get excited right away” may be related to the impulsiveness that is inherent in FS, it should be noted that this reaction might be the affective consequence of reward expectation rather than action tendencies that motivate the individual to immediate behaviors. Therefore, item 10 would be a more proper statement for BASR than for BASF, consistent with the original English version.

#### BASF

The Fun Seeking subscale was supposed to measure the instantaneous desire to approach potential or new rewards (33). The FS items in our sample showed either weak loadings on FS or cross-loadings on the RR and Drive (even BIS) items. A previous study found that FS did not enter into the best-fit model as did two other subscales of BAS (Reward Responsiveness and Drive) when the convergent validity of currently available RST questionnaires was examined (34). One possibility is that the content of the FS subscale, which focuses on fun activities, may influence the detecting power in different age groups (from 16 to 54) (34). Specifically, BAS and BIS scores increased with age in young adulthood, peaked at 20–25 years old and subsequently declined into later adulthood (49). The same reason may restrict the FS subscale from surviving the model fit criterion in our sample, which spanned 18–30 years of age. In support of the age effects, our preliminary study yielded different model structures between our two age groups (18–30 and 30–40 years) (data not published). Because the sample size of the age group ranging from 30 to 40 years was relatively small, we reported only the age group ranging from 18 to 30 years. However, the validation of age effects necessitates a larger military sample with the full age range of adulthood. Moreover, the need for social desirability may impede participants from disclosing their impulsive dispositions (detected with FS), which are typically associated with externalizing behaviors (54). This outcome could be more apparent in a collectivistic culture, which encourages social affiliation and leads to lower acceptability of externalizing behaviors (55). We strongly recommend that future studies examine the wording of the Fun Seeking subscale and other problematic items.

#### BASD

Across the two models of Chinese samples (undergraduate and military 4-factor), the items pertaining to the Drive subscale remained unchanged. The conceptualization of Drive (persistent pursuit of goals) (33) primarily relates to executing a plan of behavior, which was one of the sub-goal processes of BAS (56). Item 16 (“When I go after something I use a ‘no holds barred’ approach”) and item 8 (“If I see a chance to get something I want, I move on it right away”) were included in BASD in the Chinese revised BBS, whereas they were reassigned to the BASF and BASR subscales in our sample. By contrast, item 7 (“I'm always willing to try something new if I think it will be fun”) belonged to BASF in the original and Chinese revised BBS, whereas it belonged to BASD in our sample.

#### BIS

In contrast to the Chinese undergraduate group and Carver's original model, item 13 (“When good things happen to me, it affects me strongly”) was previously grouped into the RR subscale. While the original wording of item 13 depicted emotional responses elicited by external events, the translated item seemingly depicted stronger reactions than merely emotional. From the perspective of our participants, “good things” may seem anxiety-provoking or conflicting and thus be resolved by the BIS system until behavioral resolution occurs in favor of approach or avoidance (12).

### Demographic Variables Related to High Avoidance Motivation and Low Approach Motivation

Our results extend previous evidence that demographic variables (gender, family style, education years, and age) are related to specific profiles of BAS and BIS sensitivities. First, gender differences with respect to reward and punishment conditioning will affect the sensitivity to punishment and reward in socialization contexts. Specifically, gender role expectations might account for the gender difference in BIS levels (57). Higher activity output could be deemed more appropriate behavior for males, and avoidance and pessimistic behavior could be regarded as more suitable for females. Notably, BIS sensitivity was more strongly related to unpleasant affects among men than among women (58). Therefore, the mental health conditions of male military members with stronger avoidance motivational dispositions deserve our concern.

Second, ample evidence shows that interactions between child temperament and the family environment could predict later social outcomes, but scarce evidence has been detected under the RST framework. The family breeding style of a single parent may cause BIS-sensitive people to exhibit avoidance behaviors and a great increase in anxiety (activated unpleasant affect) during stress. Consequently, behavioral inhibition could discourage these children from interacting with the environment, predisposing them to isolation, social anxiety, and depression (59). Notably, higher levels of fun seeking tendencies in individuals with a single parent may lead to a higher risk of these individuals developing externalizing problems, such as higher alcohol consumption (60), in which approach or reward-seeking behaviors become dysregulated. Furthermore, our findings emphasize the impact of other mental health problems (especially obsessive-compulsion) on motivational dispositions, which are consistent with a previous study (61) indicating the relationship between impulsivity traits and different types of obsessive-compulsion.

Third, more education years are related to high BASD, BASR, and BASF scores, but not to BIS levels. BASD and BASR correspond to the appetitive and consummatory stages of reward processing. Persons with BAS sensitivity are more reactive to incentive signals and inclined to initiate goal-directed behaviors, which are required by higher education. Presumably, since higher levels of goal-conflict detection (one of the functions of BIS) debilitate career planning (62) and the personality factor of goal-drive persistence (BASD) is closely related to career motivations, education status may be an influential factor for career motivations and planning among military servicemen.

Finally, younger people tend to score higher on BASD and BASF. This finding means that young military personnel strive to pursue pleasurable stimuli and show a heightened response to positive or reward-laden cues (5). It is intriguing that officers and soldiers tend to have greater BASR levels than sergeants, which suggests a more thorough examination into the cause of this phenomenon.

### Relationship Between Approach/Avoidance Motivation and Mental Disorders

Collectively, depression and PTSD significantly predicted BIS and BAS levels, while the results for anxiety and OCD depended on specific conditions. First, depression showed main effects on both BASF and BIS, which echoes the theory that approach deficits (BASF and BASD) and avoidance motivation (BIS) play a role in reducing positive experiences and reinforcement for non-depressed behavior, contributing to the onset and maintenance of depression.

Second, despite a lack of main effect of anxiety on any one of the BIS/BAS subscales, the comorbidity of PTSD and anxiety significantly predicted BIS levels, indicating that individuals with low PTSD and low anxiety and those with high PTSD and high anxiety are more inclined to exhibit greater BIS sensitivity. Additionally, anxiety was related to BIS under the former condition. Our research replicates previous findings linking high BIS sensitivity to anxiety (27, 63), but only for individuals with low to medium severity of anxiety symptoms (indicated by correlations) and anxious individuals with comorbid PTSD (indicated by simple effect). Consequently, our results emphasize the importance of BIS sensitivity as a significant predictor for anxiety-related disorders.

Third, despite the lack of main effects of obsessive-compulsive symptoms on the constellations or each single BIS/BAS subscale score, the correlations between obsessive-compulsive symptoms and motivation scores were significant in most cases: the symptoms tended to be more severe for individuals with stronger approach/avoidance motivation. Presumably, these correlations may be explained by the prevalent comorbidity of OCD with other mood symptoms (anxiety and depression). Our results echo previous evidence that OCD individuals demonstrated impaired motivation (e.g., behavioral inhibition) (64). As further evidence, compulsive buying, a typical obsessive-compulsive symptom, was associated with higher approach tendencies and more depressive symptoms (65). Collectively, the lack of main effects, significant interactive effects with other symptoms without accounting for PTSD symptoms, and significant correlational relations between motivation and OCD symptoms suggest that a biased approach and avoidance motivation might be sufficient to explain the variation of obsessive-compulsive symptoms, whereas the converse is not true.

Finally, PTSD symptoms yielded main effects on the constellations or each single BIS/BAS subscale score. Whereas, PTSD symptoms did not significantly correlate with motivation scores for the high-PTSD group, PTSD symptoms significantly correlated with each BBS subscale score for the low-PTSD group. Furthermore, the comorbidity of PTSD and depression significantly predicted BASR levels. The effects of PTSD symptoms on BAS levels were confirmed according to the logistic regression analysis. Therefore, PTSD symptoms may be causally related to both approach and avoidance motivation deficits.

### ER as a Mediator Between Reinforcement Sensitivity and Internalizing Symptoms

As predicted, immersion could exacerbate the impact of BAS/BIS sensitivities on depressive/PTSD symptoms. Immersion is a type of ER strategy that involves viewing memories through one' s own eyes and experiencing self-relevant events and emotions in the first person (66). This strategy is the opposite of self-distancing, which refers to reflecting on negative experiences from the observer's perspective (67). In addition, immersion is different from rumination, one response style, which involves “repetitively focusing on the fact that one is depressed; on one's symptoms of depression; and on the causes, meanings, and consequences of depressive symptoms” (68). Rumination is deemed a mental habit triggered by the context independent of goals and is resistant to change (69), while immersion is in service of instrumental goals to modify one's emotional responses (69). Similar to rumination, immersion is an ineffective ER strategy that is relevant to prolonged emotional episodes (70), which is typical of emotional disorders such as depression.

Reinterpretation, one of the most studied reappraisal strategies, involves changing the meaning of a stimulus (71). Compared with expression suppression, reinterpretation successfully alleviated the impact of BAS sensitivities on depressive/PTSD symptoms. These results were consistent with a previous meta-analysis, which suggested that reinterpretation is more advantageous than expression suppression in regulating emotion (72). Consistent with this evidence, the transformation of negatively biased interpretations is actually one of the primary goals of cognitive therapy for depression.

Talking out could alleviate the impact of BAS/BIS sensitivities on depressive/PTSD symptoms. Talking out is one of the less-explored strategies in experimental studies, possibly due to the difficulty in manipulating the extent and content of this type of ER. Notably, only talking out is successful at alleviating the impact of BIS sensitivity on depressive/PTSD symptoms, which emphasizes the importance of psychological assistance rather than skill training for individuals with stronger avoidance motivation.

Collectively, these results suggest that immersion may be an ineffective ER strategy for mood disorders, whereas reinterpretation and talking out may be effective ER strategies for mood disorders (Box 1). Because ER strategies also have influential indirect effects on motivation and mood symptoms, our results underscore the importance of ER as a specific target for psychotherapy directed at motivation (73).

Box 1 **Take-home message**

The validity and reliability of the four-factor model (BIS, Drive, Reward Responsiveness, and Fun Seeking) of BBS in Chinese Military men were supported.

Gender (female), family status (single-parent family), and social relationships (having fewer good friends) were significant predictors of high BIS sensitivity.

More education years, greater age, being an only child and family status (single-parent family) significantly predicted high BAS sensitivity.

BIS/BAS scores could predict the symptoms of depression, anxiety, OCD, and PTSD.

While immersion exacerbated the impact of BAS/BIS sensitivities on depressive/PTSD symptoms, reinterpretation and talking out alleviated the impact of BAS/BIS sensitivities on these symptoms.

## Limitations and Implications

Several limitations of the current study should be considered. First, the research design was cross-sectional, which prevented the identification of any causal relations among variables. However, the mediating model may provide potential predictors of specific mood symptoms (e.g., depression, PTSD). Second, the current study was conducted on a sample of participants who did not meet the criteria for current mood or anxiety disorders. Therefore, the proposed models may not be adequately applicable to clinically depressed, anxious or PTSD samples. However, epidemiological research, which could be compared with models drawn from Chinese clinical samples in future studies, provides a more conservative methodology to test the BIS/BAS model than clinical studies (17). Third, recent advances in the neuroscience of BAS have identified four distinctive aspects of BAS: wanting, incentive motivation, striving and liking (74). Thus, whether BAS scales (dispositional level) reflect individual differences in the neural substrates of these subdimensions warrants further study.

These limitations notwithstanding, the current study is unique because it establishes additional evidence for the psychometric properties of BBS in a non-Western society. The profiles of motivational dispositions might be promising biomarkers for preventative intervention of mood disorders. Moreover, ER strategies as a partial mediator for individuals with specific motivational disposition profiles are promising potential targets for future psychotherapies.

## Author Contributions

RZ, KL, LW, JZ, and XL collected the data. XW, RZ, and XC analyzed the data. XW wrote the paper. ZF designed the research and provided writing assistance of the article.

### Conflict of Interest Statement

The authors declare that the research was conducted in the absence of any commercial or financial relationships that could be construed as a potential conflict of interest.
